# X-ray microtomography is a novel method for accurate evaluation of small-bowel mucosal morphology and surface area

**DOI:** 10.1038/s41598-020-69487-w

**Published:** 2020-08-04

**Authors:** Johannes Virta, Markus Hannula, Ilmari Tamminen, Katri Lindfors, Katri Kaukinen, Alina Popp, Juha Taavela, Päivi Saavalainen, Pauliina Hiltunen, Jari Hyttinen, Kalle Kurppa

**Affiliations:** 1 Center for Child Health Research, Faculty of Medicine and Health Technology, Tampere University, Arvo Ylpön katu 34, 33520 Tampere, Finland; 2 Department of Pediatrics, Tampere University Hospital, Tampere, Finland; 3 Computational Biophysics and Imaging Group, The Faculty of Medicine and Health Technology, Tampere University, Tampere, Finland; 4 Celiac Disease Research Center, Faculty of Medicine and Health Technology, Tampere University, Tampere, Finland; 5 Department of Internal Medicine, Tampere University Hospital, Tampere, Finland; 6 Carol Davila University of Medicine and Pharmacy, and Alessandrescu-Rusescu National Institute for Mother and Child Health, Bucharest, Romania; 7 Central Finland Central Hospital, Jyväskylä, Finland; 8 Department of Medical and Clinical Genetics, University of Helsinki, Helsinki, Finland; 9 The University Consortium of Seinäjoki, Seinäjoki, Finland

**Keywords:** Biophysics, Biotechnology, Gastroenterology

## Abstract

The often poorly orientated small-bowel mucosal biopsies taken for the diagnostics of celiac disease and other intestinal disorders are prone to misinterpretation. Furthermore, conventional histopathology has suboptimal sensitivity for early histopathological changes observed in short-term challenge studies. X-ray microtomography (micro-CT) is a promising new method for accurate imaging of human-derived biological samples. Here, we report that micro-CT could be utilized to create virtual reconstructions of endoscopically obtained intestinal biopsies. The formed digital 3D images enabled selection of always optimal cutting angles for accurate measurement of the mucosal damage and revealed diagnostic lesions in cases interpreted as normal with conventional histomorphometry. We also demonstrate that computer-assisted point cloud analysis can be used to calculate biologically meaningful surface areas of the biopsies in different stages of mucosal damage with excellent replicability and correlation with other disease parameters*.* We expect the improved diagnostic accuracy and capability to measure the surface areas to provide a powerful tool for the diagnostics of intestinal diseases and for future clinical and pharmaceutical trials.

## Introduction

Correct identification of celiac disease is essential, as misdiagnosis may lead to morbidity related either to ongoing duodenal damage or to unnecessary dietary restriction. The diagnosis has traditionally been based on the histopathologic evaluation of small-bowel mucosal biopsies. Since this is conducted on cross-sectional cuttings of the original 3D sample, precise orientation of the specimen is critical, but often difficult (Supplementary Fig. 1). Further challenges are caused by patchiness of the lesion and significant intra- and interobserver variation in the grouped histopathology routinely applied^1–4^. There is a preference for a less invasive diagnostic approach, at least in Europe, but the biopsy remains obligatory in adults and in children with low positive celiac serology, i.e. in the diagnostically most challenging cases^5,6^. Besides the diagnosis, improved histological assessment would be welcomed for pharmaceutical studies^7^.

An interesting new imaging method for biological samples is X-ray microtomography (micro-CT). Examination of the small intestine can nowadays be done with conventional CT, but micro-CT imaging for human derived biopsies has not yet been tested^8–10^. Micro-CT enables virtual 3D modeling and cross sectioning of the original specimen at arbitrary angles without destroying it^11,12^. The often encountered low tissue contrast can be improved by contrast enhancement reagents^13,14^. The generated 3D image allows free rotation and digital cutting, which, in the case in small-bowel mucosal samples, could enable optimal orientation of the villi for exact morphometry. It might even be possible to measure surface areas for more sensitive and biologically meaningful estimations of the mucosal health. Moreover, we hypothesized that, if successful in paraffin-embedded biopsies, micro-CT could be used to re-evaluate archived samples taken even years earlier. However, micro-CT imaging has not so far been tested with human-derived intestinal samples.

We investigated whether micro-CT imaging could be utilized to improve the accuracy of the morphometric analyses of small-bowel mucosal biopsies and with distinguishing between healthy and diseased states, particularly in subjects with suspected celiac disease. Additionally, as a completely novel approach to attain precise information of the mucosal changes, we explored the possibility of performing surface area measurements from the digitalized biopsies.

## Materials and methods

### Patients and study design

The study was carried out at Tampere University and Tampere University Hospital. Biopsies for micro-CT imaging were selected from among samples taken from individuals who had undergone esophagogastroduodenoscopy with duodenal sampling and who had given their consent to participate in research projects. The selected subjects represented different stages of small-bowel changes from morphologically normal villi to severely diseased mucosa, as determined in conventional histology. All subjects with positive celiac disease serology started a gluten-free diet, either as a treatment after diagnosis or as an experimental trial^15^. Besides the endoscopy, supplementary laboratory measurements, including measurement of celiac disease serology and genetics, were carried out. Repeat biopsies were obtained after approximately one year on a gluten-free diet. The diagnosis of celiac disease was based on the detection of small-bowel mucosal atrophy^16^. The Regional Ethics Committee of Pirkanmaa Hospital District approved the study protocol and patient recruitment. All participants gave written informed consent. Patients or the public were not involved in the design, or conduct, or report or dissemination of our research. All experiments were performed in accordance with relevant guidelines and regulations.

### Celiac disease serology and genetics

Serum autoantibodies to transglutaminase 2 (TG2ab) were measured by commercial ELISA assay (Phadia AB, Uppsala, Sweden) with values ≥ 5.0 U/ml considered positive. Serum endomysium antibodies (EmA) were measured by indirect immunofluorescence using human umbilical cord as a substrate^17^. A titer of 1: ≥ 5 for EmA was considered positive and further diluted up to 1:4,000 until negative. Celiac disease-associated gene alleles encoding HLA-DQ2 and DQ8 were analyzed as described elsewhere^15,18^.

### Endoscopies, histopathology, and immunohistochemistry

All esophagogastroduodenoscopies were performed using a standard endoscope (Olympus Corp., Tokyo, Japan) and routine sampling techniques. At least four forceps biopsies were systemically obtained from the duodenum and referred for conventional histopathologic analysis. In addition, several biopsies were taken for research purposes. Processing of the research biopsies was carried out according to their further use as described below.

**Figure 1:**
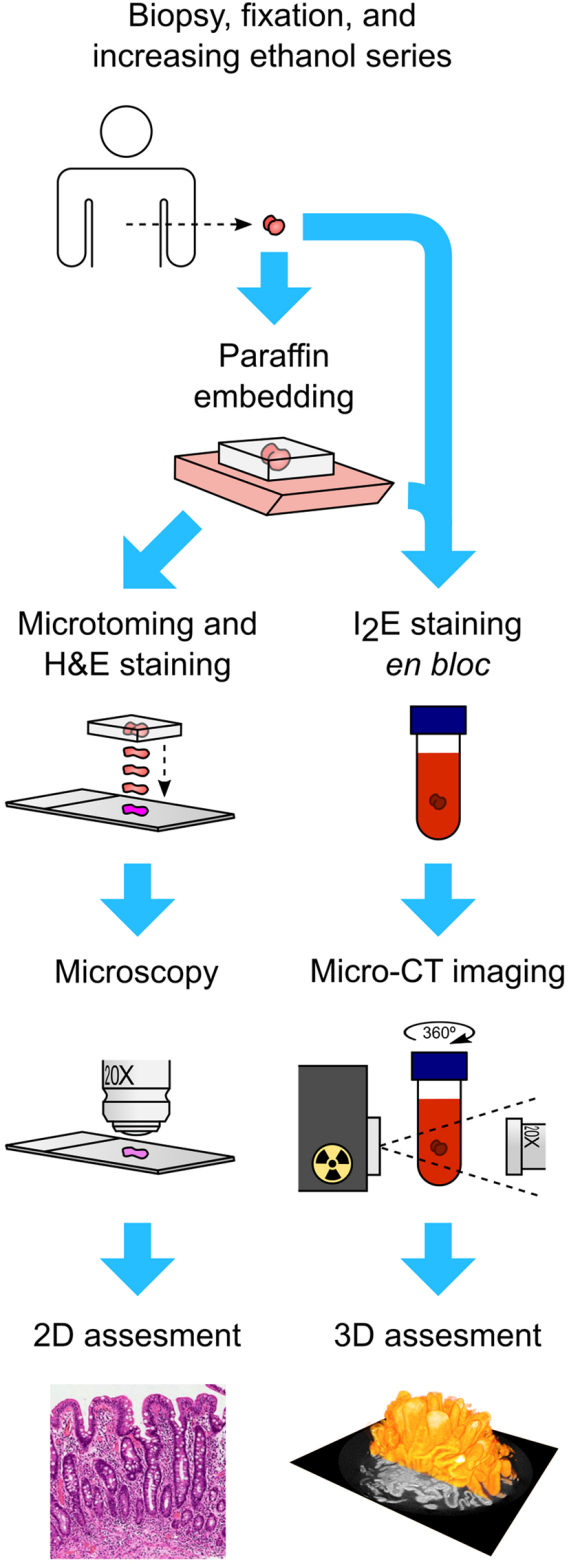
Comparison of conventional histology and micro-CT imaging of the small-bowel mucosal biopsies. After sampling, the specimen is fixed with formalin and dehydrated with increasing ethanol series. Next, in conventional histology (left route), the biopsy is embedded in paraffin via xylene and then cut into thin sections, stained with H&E, and evaluated under light microscopy. The assessment is performed either applying grouped classification^42^ or by quantitative determination of the villous height-crypt depth ratio^3^. Correct orientation of the biopsy is a prerequisite for reliable measurements. This is accomplished by recutting the sections until acceptable readouts are obtained, assuming that the biopsy size is sufficient for this. Micro-CT imaging (right route) starts by placing fixed and ethanol-dehydrated biopsies with or without paraffin embedding into I_2_E solution to increase the intrinsically low soft tissue contrast. Subsequently, mechanically stabilized biopsy is scanned using X-ray source and detector, and the acquired transmission images are reconstructed to 3D model. The model is freely orientable for precise morphometric measurements and also enables measurement of the mucosal surface areas.

In addition, histology was assessed with validated histomorphometry^3^. The biopsies were formalin-fixed, embedded in paraffin conventionally via increasing ethanol-dehydration series and xylene, cut in 2 µm sections, and stained with hematoxylin–eosin (H&E) (Fig. 1). Special attention was paid to the correct orientation and only sections containing longitudinally cut crypts were accepted for further analyses. Recuttings were requested until apparently acceptable morphometric readouts were obtained. Quantitative villous height/crypt depth ratio (VH:CrD) was analyzed as an average of at least three individual crypt-villous pairs, considering a VH:CrD < 2.0 diagnostic for celiac disease^3^.

A subset of the duodenal biopsies was snap-frozen and used for immunofluorescent determination of small-bowel mucosal TG2-targeted immunoglobulin A (IgA) deposits. After sampling the biopsies were freshly embedded in optimal cutting temperature compound (Tissue-Tec, Miles, Elkhart, IN) and snap-frozen in liquid nitrogen. The deposits were investigated from 5 µm thick frozen sections by double-labeling TG2 and IgA as previously described^16^. The deposits have been shown to have an excellent specificity for celiac disease even before manifest morphological lesion^19^.

### Processing and micro-CT imaging of the duodenal biopsies

Before micro-CT imaging, excess paraffin was removed from the paraffin-embedded biopsies around the actual tissue, and the remaining sample was placed in contrast-enhancing iodine-based solution (I_2_E) (Fig. 1)^20^. The solution was made by dissolving solid iodine (207772, Sigma Aldrich, MO) in absolute ethanol to achieve a concentration of 10 mg/ml. Solution times of 3–25 h were tested to ensure sufficient saturation for optimal contrast enhancement throughout the sample. Next, the samples were placed in 1 ml plastic syringe and stabilized between rubber pistons. The syringe was filled with the I_2_E to preserve the chemical conditions and to eliminate outward diffusion of the contrast agent. Chemical and mechanical stability of the sample was monitored by collecting a set of images (drift file) from a fixed angle during imaging. In order to test for possible faster tissue saturation, a subset of duodenal biopsies were imaged without paraffin embedding and, instead of the xylene step, by transferring the samples directly to the I_2_E.

All micro-CT images were acquired by MicroXCT-400 device (Xradia, Carl Zeiss AG, CA). The protocol was optimized by testing X-ray acceleration voltages of 40 kV, 60 kV, 80 kV and 100 kV with and without filtering, aiming to achieve optimal image resolution and contrast within a practical period of time. Image quality was assessed visually as in standard histopathology. To create 3D images, the data were reconstructed by XMReconstructor 8.1.6599 software (Xradia). Avizo software (Thermo Fisher Scientific, Waltham, MA) was used to produce example videos of the digital cutting and morphometric evaluation of the 3D images.

### Surface area measurements

The surface area of a selected 3D biopsy model was measured by applying computer-assisted point cloud analysis with Avizo software (Thermo Fisher Scientific, Waltham, MA). First, the 3D image was denoised to refine surface of the sample for the point cloud analyzes^21^. According to out prior experience Non-local filter was used for this task. Median filter was also tested but it did not provide additional benefits over Non-local filter. Then the tissue area was segmented from the background using manual thresholding. Next, analysis of the effective surface area was carried out by aligning a rectangle of predefined size with the mucosa and perpendicular to the villi. The point cloud of the tissue surface provided by segmentation was used to calculate the surface within these boundaries. The surface area was calculated using discretized generalization of the Crofton formula to 3D point cloud (Fig. 4)^22^. The measured area was compared with that of an equal but completely flat rectangle according to the following equation:

$$ Effective\;surface\;area = \frac{{Measured\;surface\;area\;from\;selected\;rectangle \left( {{\text{mm}}^{2} } \right)}}{{Area\;of\;selected\;rectangle \left( {{\text{mm}}^{2} } \right)}} $$

Next, the result was given as a coefficient to this theoretical minimum value of 1.0.

In order to evaluate the optimal margins and replicability of the measures, squares with increasing side lengths from 0.1 to 1.0 mm at every 0.1 mm were tested by measuring 20 individual measurements from the same biopsy and counting the means and 95% confidence intervals. This was done in three separate biopsies representing severe mucosal damage, less advanced lesion and normal morphology based on VH:CrD (Supplementary Fig. 2).

## Results

### Patient characteristics and histomorphometry

The study comprised eight subjects who had undergone duodenal sampling due to various symptoms and signs; the six cases with positive celiac autoantibodies also underwent repeat biopsy after approximately 1 year on a gluten-free diet (Table 1). After the conventional histopathology with quantitative morphometry, three subjects received a celiac disease diagnosis, three had potential celiac disease with positive TG2ab and EmA and VH:CrD > 2.0, and the two seronegative subjects had normal villi (Table 1). All seropositive cases had celiac disease-associated HLA-DQ2/DQ8 and positive mucosal IgA deposits, and demonstrated clinical and serological response to the dietary treatment (Table 1). One of the seronegative subjects had HLA-DQ2 and the other was negative for both predisposing alleles. Duodenal histomorphometry improved in all celiac disease patients on a gluten free diet. Furthermore, despite the allegedly normal villi at baseline, VH:CrD also increased in cases with potential celiac disease (Table 1).

**Table 1:** Clinical, serological and histological characteristics of the study patients.

No:	Age, years	Sex	Indication for endoscopy	EmA, titer	tTGab, U/l^a^	Histomorphometry		Micro-CT imaging		
						VH/CrD	Diagnosis	VH/CrD	Surface area^b^	Diagnosis
1	67	F	Diarrhea, bloating	1:1,000	42.1	0.1	CD	0.8	2.8	CD
	68		Repeat biopsy on a GFD	Negative	3.0	2.5	Healed CD	2.2	6.0	Healed CD
2	45	M	Protein-losing enteropathy	1:4,000	> 100.0	1.3	CD	0.5	1.8	CD
	46		Repeat biopsy on a GFD	1:200	26.4	2.6	Healed CD	2.1	6.1	Healed CD
3	68	F	Unexplained anemia	1:200	89.8	0.1	CD	0.1	1.3	CD
	69		Repeat biopsy on a GFD	Negative	2.4	0.4	Improved CD	0.9	3.3	Improved CD
4	39	M	Diarrhea, bloating	1:5	7.7	2.2	Potential CD	1.0	2.1	CD
	40		Repeat biopsy on a GFD	Negative	0.3	4.3	*Healed CD?*	2.7	5.9	Healed CD
5	46	F	Abdominal pain, bloating	1:5	4.6	2.3	Potential CD	1.3	3.2	CD
	47		Repeat biopsy on a GFD	Negative	0.6	2.8	*Healed CD?*	2.0	5.6	Healed CD
6	44	F	Diarrhea, abdominal pain	1:50	6.8	3.0	Potential CD	2.6	3.8	Potential CD
	45		Repeat biopsy on a GFD	Negative	2.1	3.3	*Healed CD?*	2.9	5.9	*Healed CD?*
7	65	F	Dyspepsia	Negative	0.6	2.8	No CD	2.6	4.4	No CD
8	53	M	Dyspepsia	Negative	0.8	2.8	No CD	2.5	5.9	No CD

*CD* celiac disease, *EmA* endomysium antibodies, *GFD* gluten-free diet, *tTGab* tissue transglutaminase antibodies, *VH/CrD* villous height crypt depth ratio.

^a^Reference value < 5.0 U/l.

^b^In relation to the theoretical minimum value of 1.0 of perfectly flat mucosa.

### Micro-CT imaging

Sufficient tissue saturation was achieved when the paraffin-embedded biopsy was placed in the I_2_E solution for 12 h. Both whole and partially microtomed paraffin biopsies could be utilized. In order to achieve complete I_2_E saturation, excess paraffin had to be removed from around the samples. Although saturation could theoretically be faster without paraffin embedding, there was no significant difference in image quality between biopsies with and without paraffin after contrast enhancement. In fact, the paraffin-stabilized samples showed sample-movement artifacts less often, and were thus used for the study. It was also possible to conduct conventional histopathology after micro-CT imaging, although the samples were more brittle due to the I_2_E treatment.

**Figure 2:**
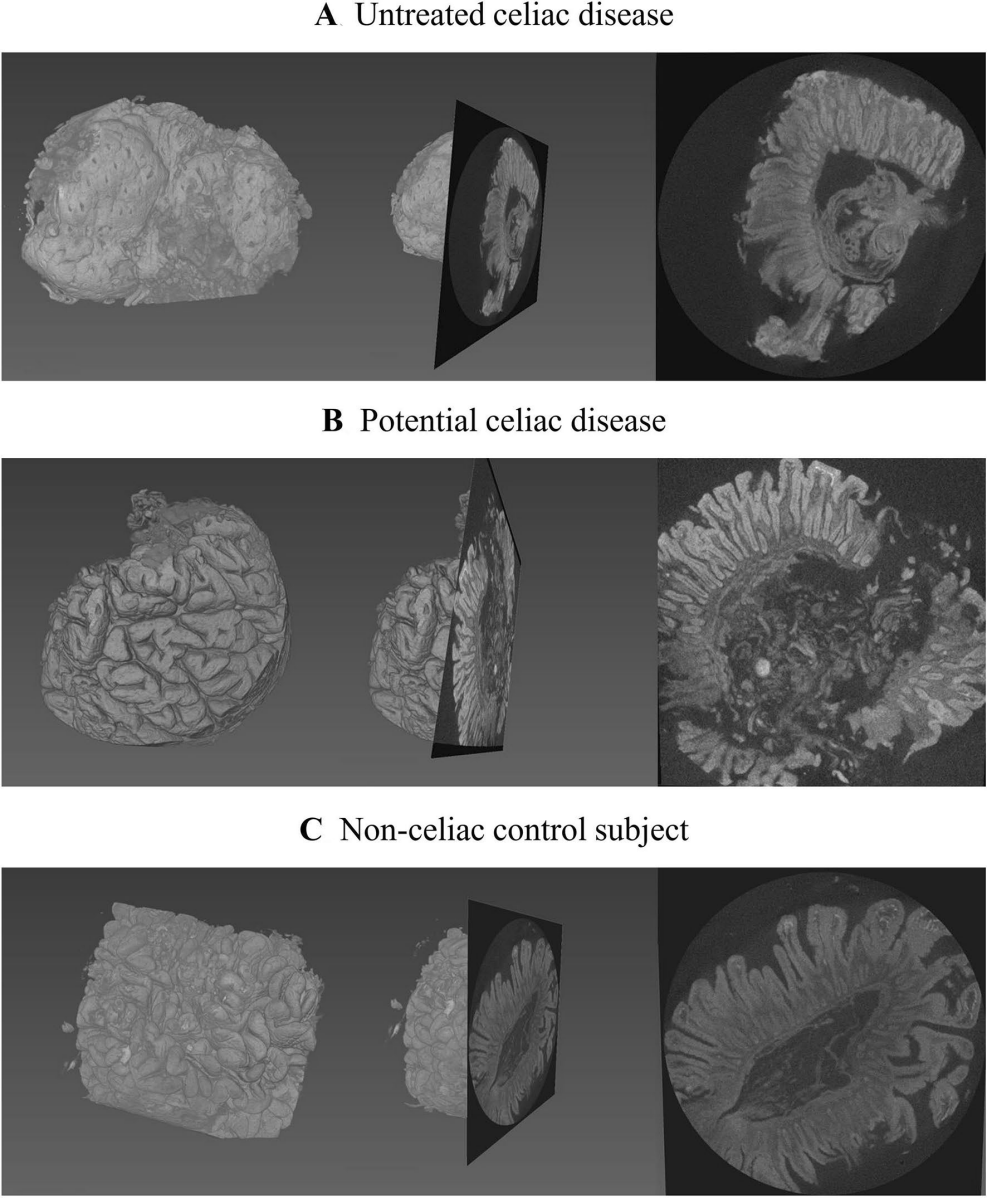
Examples of 3D biopsy reconstructions and digitally cut sections from an untreated celiac disease patient diagnosed in routine practice (**A**), from a seropositive individual with duodenal morphology interpreted as normal (potential celiac disease, **B**), and from a non-celiac control subject with negative serology and normal villi (**C**). The image scaling is uniform throughout the figure. The 3D model allows free virtual orientation and rapid setting of an optimal cutting angle for precise digital morphometry. Although the biopsy in Panel B was originally interpreted as normal, possibly due to incorrect orientation or patchy mucosal lesion, the abnormal surface structure with widened and blunted villi can be readily seen in the 3D reconstruction; particularly when comparing with the longer villi in (**C**). The orientated digital section on the right side of the (**B**) confirms the decreased villous-crypt ratio characteristic of celiac disease. (**C**) Also demonstrates the variability in the villous morphology even in healthy mucosa. The structural differences seen in the middle of the image (right side of the panel) is only random variation that has no diagnostic significance.

After optimization of the procedures, we decided to use non-filtered X-ray radiation with 100 kV acceleration voltage and 10 W source power for the imaging. The chosen 100 kV provided clearly sufficient quality with a reasonable imaging time. Despite the relatively high voltage, no beam hardening was observed in the 3D reconstructions. Altogether 1,600 X-ray projections were evenly acquired 360° around each sample with 5 s exposure time per image. Use of X-ray detection scintillator with 10 × objective and binning 2 gave a voxel size of approximately 2 μm. The subsequently reconstructed 3D digital biopsies accurately showed the general appearance and main tissue features of the duodenal mucosa (Fig. 2).

Furthermore, the 3D biopsies could be freely manipulated and digitally cut back into slices with optimal plane of viewing for precise morphometric measurements of the villi and crypts (Fig. 2, Video 1, Video 2).

### Comparisons between conventional histology, histomorphometry, and micro-CT

**Figure 3:**
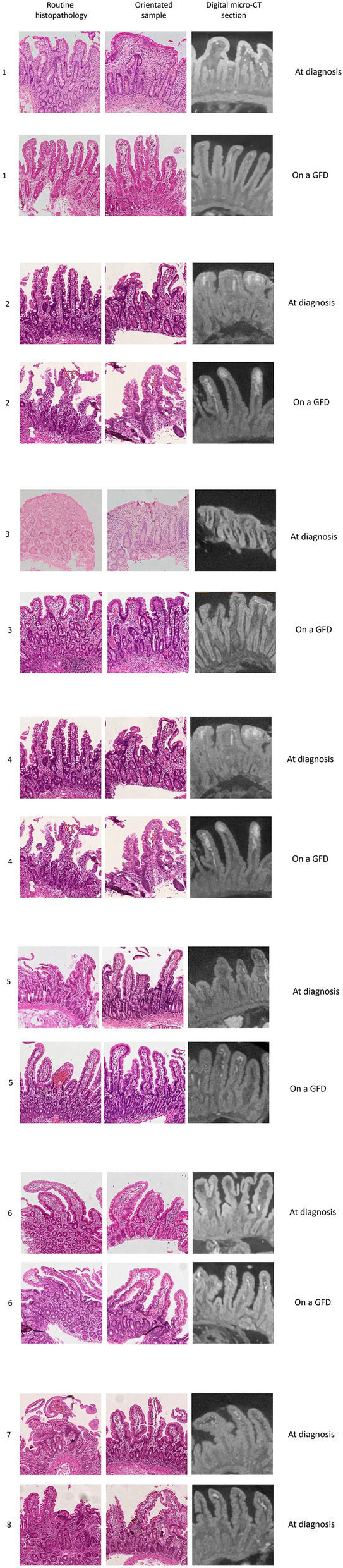
Duodenal biopsies evaluated in routine practice with conventional histology (photos on the left), orientated sections investigated by quantitative histomorphometry (middle photos) and digital cuttings obtained via reconstructed micro-CT models (photos on the right). In patients 1–6 biopsies were also taken after approximately one year on a gluten-free diet (GFD). Patients 1–3 had received a celiac disease diagnosis, subjects 4–6 had positive celiac disease serology but were originally considered to have normal villi (potential celiac disease) and subjects 7–8 exemplify non-celiac controls with negative serology and normal histology. Numbering of the patients and samples corresponds Table 1.

Figure 3 shows the H&E stained sections used for routine histopathology, the corresponding cuttings evaluated by quantitative morphometry, and the digital sections obtained by micro-CT. The figure demonstrates the frequently poor biopsy quality and incorrect cutting angle in the routine sections, revealed by circular sections of the mucosal crypts. Utilization of these sections in the diagnostics increases the risk of misinterpretation and they should not be used for diagnostic evaluation*.* Images in the middle sections of Fig. 3 are clearly better orientated with the longitudinally cut crypts indispensable for accurate histomorphometry^3^. However, achieving these results required several laborious re-evaluations and, in fact, in many cases remained suboptimal, as it was impossible to further recut biopsies of limited size. Patchiness within the biopsies and difficulties in acquiring evenly distributed longitudinal cuttings of the crypts further hampered the analysis of borderline cases, particularly as it was often impossible to further recut biopsies of limited size.

The 3D micro-CT reconstructions of the biopsies allowed easy and accurate digital sectioning in each case (Fig. 3). The resulting improved diagnostic accuracy actually revealed a clear celiac-type lesion in two patients, as well as their morphometric healing on GFD (cases 4 and 5 in Fig. 3). Furthermore, the orientation of the individual villi varied within a single biopsy, and to make truly precise measurements each villus needed to be cross-sectioned separately instead of a singular plane of view, which was possible only with micro-CT reconstructions. The resolution was somewhat lower than with H&E stainings, but sufficient for the determination of VH/CrD. Micro-CT analysis changed the result in seven subjects; in all but one to a more severe lesion (Table 1). Moreover, in accord with the clinical, serological and histological response to gluten-free diet, two cases with potential celiac disease in conventional histology showed diagnostic lesion in digital cutting (Table 1, Fig. 3). The general appearance of surface of the 3D biopsy models obtained from these subjects was already markedly different from that of the non-celiac controls (Fig. 2). The diagnosis of one seropositive subject (n:o #6) did not change even after micro-CT evaluation (Table 1).

### Surface area measurements

**Figure 4:**
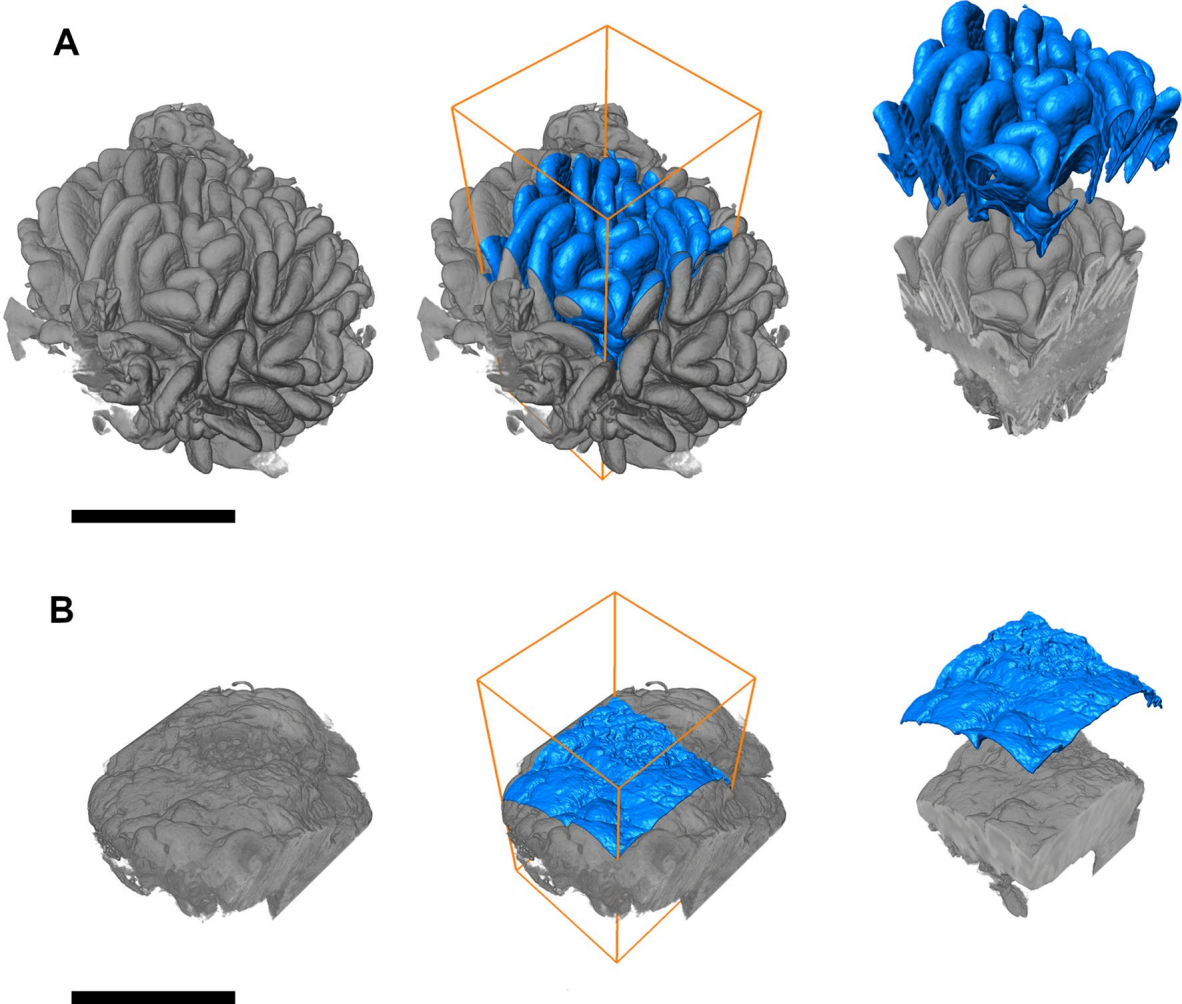
Demonstration of the surface area measurement from the digital 3D models of the small-bowel mucosal biopsies by computer-assisted point cloud analysis (Avizo software; Thermo Fisher Scientific, Waltham, MA, USA). The image scaling is uniform throughout the figure. Panel (**A**) represents morphologically normal mucosa from a non-celiac individual and Panel (**B**) advanced celiac disease with almost flat mucosa. For the measurements, a rectangle of pre-defined size is aligned with the mucosa and the surface area is calculated within these boundaries. It is possible to use different rectangular side lengths and various measurement shapes. Use of equal image resolution and noise filtering is required for consistent results. The surface area in Panel (**A**) was 5.973 mm^2^ and in Panel (**B**) 1.548 mm^2^ when applying a measurement rectangle with an area of 1.000 mm^2^. Black bar = 1.0 mm.

The surface area measurements were taken from each 3D biopsy model using rectangles of different size up to 1.00 mm^2^, if feasible, excluding areas with artifacts e.g. due to biopsy handling (Fig. 4). It was also possible, for example, to use a circular measurement area but, for the sake of consistency rectangular selection was used with all samples. Regardless of the degree of mucosal damage, measurement rectangles with different basal areas provided consistent effective surface areas when applied to the same biopsy with side lengths ≥ 0.5 mm; with lengths < 0.5 mm uncertainty and differences from larger measurement sizes began to emerge and the selection of the mucosal site became more significant (Supplementary Fig. 2). Alignment of the measurement rectangle was easy and replicable (data not shown).

There was a logical relationship between the surface areas and corresponding VH:CrD (Table 1). More specifically, with a flat mucosa the surface area coefficient approached the theoretical minimum of 1.0, while the corresponding values of non-celiac controls and patients with a healed mucosa were 4.4–6.1. Furthermore, all cases with potential celiac disease in conventional histology, including case #6 with a normal VH:CrD even in the micro-CT evaluation, showed reduced surface area at baseline, and a subsequent increase of the area to the level of controls and cured patients on treatment (Table 1).

## Discussion

We succeeded in developing a practical micro-CT imaging protocol for virtual 3D reconstruction of human-derived intestinal biopsies. The method enabled fast and replicable digital cutting of the biopsies for precise digital morphometry. Notably, the amount of human labor required for the morphological measurements from 3D images was only a fraction when compared with the average of standard histomorphometry. We believe that the novel approach can significantly improve the diagnostics of celiac disease, which currently involves many challenges^23^. Particularly difficult in conventional histopathology is, as also demonstrated here, correct sectioning of the biopsies for accurate microscopic evaluation^3,4,24^. This is one of the main reasons why, even if quite replicable with normal villi and flat mucosa, traditional grouped classification exhibits substantial intra- and interobserver variations between these extremities^25,26^. Due to increasing screening, such cases with inconclusive histology are becoming more prevalent^2,15,27^. Quantitative morphometry may be more accurate in these circumstances, as special emphasis is placed on correct biopsy orientation^3^. Nevertheless, this requires expertise and, despite laborious re-cuttings, often remains suboptimal. The 3D micro-CT model can be endlessly re-orientated, and the reader can gain an impression of the variability of the villi and identify technical artifacts. As a result, even in this proof-of-concept study, we were able to identify celiac disease in two subjects with non-diagnostic conventional histology. Their diagnoses were further confirmed by the presence of the correct genetics, positive mucosal IgA deposits and dietary response^15,19^.

Besides diagnostics of celiac disease, another field in which better histological tools are needed is in treatment trials. There is an increasing demand for alternatives to the burdensome gluten-free diet, and accurate outcome measures are critical for the required trials^7,28^. Histology remains the gold standard, as mucosal healing is a prerequisite for a treatment to be effective, and the current non-invasive surrogate markers lack the necessary sensitivity^7,28,29^. Recently developed innovative immunological outcome measurements, such as HLA-DQ-gluten tetramer test^30^, are promising but require further validation^7^. The problem with conventional histology is, again, the poor geometrical accuracy and replicability, particularly with the minor changes seen during short-term gluten challenge studies. This is the case even with morphometry, as the early damage is often patchy and variable even within the same biopsy^7^. The situation differs from diagnostic evaluation, in which the most damaged area is sought, whereas in prospective studies replicability of the histology is more important. This further emphasizes the benefits of micro-CT, as the correct cutting angle can be selected individually from several villi, which reduces the risk of random variation and inaccuracy caused e.g. by twisted biopsy.

As an entirely novel approach, we succeeded in analyzing the mucosal surface area. Although confirmation in larger series is needed, the technique seemed to offer excellent accuracy and replicability, as well as to correlate logically with other disease parameters. This, together with the small variation in the results between different measurement rectangle sizes and positions on the same biopsy, concurs with the theoretical premise that surface area is a more consistent outcome than (even optimized) cross-sectional evaluation^2^. It could also yield a more precise estimate of the intestinal absorption capacity. In fact, inaccuracy in conventional histology may explain the previously reported poor correlation between the degree of enteropathy and clinical presentation^31^. Interestingly, the surface structure seen in the digital 3D image of a patient with potential CD (Fig. 2) appears to be visually very different compared to the control patient and arouses a suspicion of mucosal damage. This perhaps diagnostically significant factor could only be detectable with a 3D image. The superior sensitivity of 3D measurement is also in agreement with the reduced surface area in the one subject who had gluten-sensitive symptoms but normal cross-sectional VH:CrD even with micro-CT. Altogether, the novel approach offers intriguing possibilities for the diagnostics of celiac disease and as a novel outcome for the abovementioned drug trials. Surface area measurement could also be utilized e.g. when investigating safe levels of residual gluten^32^, to confirm diagnosis in individuals with a self-initiated gluten-free diet and in the diagnostics of other disorders involving duodenal abnormalities^33^.

Some caution, however, is needed when interpreting the results. For example, it must be born in mind that reduced surface area is not the only reason for impaired intestinal absorption, as there may also, for example, be disturbances in the cell differentiation and vascular organization of the epithelium^34,35^. Interestingly, in the future micro-CT may also be utilized to quantify these more subtle histological changes^36^. Certain technical issues may also affect the measurements, including variable architecture of the biopsy surfaces, perhaps due to merging of the villi to form complex structures possibly displaying reduced surface area with normal VH:CrD. This phenomenon was already reported in the 1960s^37,38^, but has been largely ignored. It is currently unclear whether such changes represent variable stages of mucosal damage, or merely morphological variation along the length of the intestine and/or between individuals^37,39^. These disparities may also explain the somewhat unequal surface areas we observed between the non-celiac individuals here. Larger series of patients with diverse clinicopathologic features are needed to further elucidate these issues.

### Limitations of the study

The lower resolution of micro-CT reconstructions compared with conventional histology could theoretically affect the diagnostic accuracy, but this drawback is outweighed by the optimal biopsy orientation. We were unable to measure the degree of mucosal inflammation, which, although unspecific, is considered an integral part of the differential diagnostics of duodenal diseases^33^. This information might be obtainable utilizing X-ray contrast agents with specific ligands^40^ targeting e.g. CD3+ intraepithelial lymphocytes. As it was completely novel approach, calculation of the surface areas was done partly manually, but it should be fairly straightforward to automatize this process^41^. It was also not reasonable to calculate precise intra- and interobserver variations for micro-CT imaging in this proof-of-concept study, and these remain to be determined. Of note, the sample-specific imaging time could likely be decreased by reducing the number of projections from the 1,600 used here as recommended by the device manufacturer to ensure successful surface area analyzes. Further studies in this field should be conducted in various patient series. However, the actual imaging time is usually not a time limiting factor in clinical practice, as the results are not needed urgently and this is not an expensive labor-requiring step of the analysis. Finally, the method requires technical expertise and relatively expensive equipment, thus restricting its clinical use. Then again, it is quite easy to ship paraffin-embedded biopsies to specialized centers for micro-CT studies.

## Conclusions

The 3D micro-CT imaging of stained biopsies provided good contrast and precise morphometric measurement of the small-bowel mucosa in endoscopically obtained biopsies, readily demonstrated by the altered diagnosis of some patients already in this proof-of-concept study. The improved accuracy and replicability of the histopathological evaluation, together with the possibility to measure biologically meaningful villous surface areas, offer a powerful tool for the diagnostics of celiac disease and for future prospective trials.

## Supplementary information

Supplementary Infomation.

Supplementary Figures.

Supplementary Video 1.

Supplementary Video 2.

## References

[1] Corazza G, Villanacci V (2005). Coeliac disease. J. Clin. Pathol..

[2] Ravelli A, Villanacci V, Monfredini C (2010). How patchy is patchy villous atrophy: Distribution pattern of histological lesions in the duodenum of children with celiac disease. Am. J. Gastroenterol..

[3] Taavela J, Koskinen O, Huhtala H (2013). Validation of morphometric analyses of small-intestinal biopsy readouts in celiac disease. PLoS ONE.

[4] Werkstetter K, Korponay-Szabó I, Popp A (2017). Accuracy in diagnosis of celiac disease without biopsies in clinical practice. Gastroenterology.

[5] Husby S, Koletzko IR, Korponay-Szabó ML (2012). European Society for Pediatric Gastroenterology, Hepatology, and Nutrition guidelines for the diagnosis of coeliac disease. J. Pediatr. Gastroenterol. Nutr..

[6] Rubio-Tapia A, Hill I, Kelly C (2013). American College of Gastroenterology. ACG clinical guidelines: Diagnosis and management of celiac disease. Am. J. Gastroenterol..

[7] Ludvigsson J, Ciacci C, Green PH (2018). Outcome measures in coeliac disease trials: The Tampere recommendations. Gut.

[8] Francis JS, Jalil A, Spencer CB (2011). CT findings in adult celiac disease. Radiographics..

[9] Michael M, Alec JM, Emil JB (2007). A pattern approach to the abnormal small bowel: Observations at MDCT and CT enterography. AJR Am. J. Roentgenol..

[10] Hetal D, Scott M, Ajay S (2008). Computed tomographic enterography and enteroclysis: Pearls and pitfalls. Curr. Probl. Diagn. Radiol..

[11] Happel CM, Klose C, Witton G (2010). Non-destructive, high-resolution 3-dimensional visualization of a cardiac defect in the chick embryo resembling complex heart defect in humans using micro-computed tomography: Double outlet right ventricle with left juxtaposition of atrial appendages. Circulation.

[12] Mizutani R, Suzuki Y (2012). X-ray microtomography in biology. Micron.

[13] Jeffery NS, Stephenson RS, Gallagher JA (2011). Micro-computed tomography with iodine staining resolves the arrangement of muscle fibres. J. Biomech..

[14] de Crespigny A, Bou-Reslan H, Nishimura MC (2008). 3D micro-CT imaging of the postmortem brain. J. Neurosci. Methods.

[15] Kurppa K, Collin P, Viljamaa M (2009). Diagnosing mild enteropathy celiac disease: A randomized, controlled clinical study. Gastroenterology.

[16] Husby S, Murray JA, Katzka DA (2019). AGA Clinical practice update on diagnosis and monitoring of celiac disease-changing utility of serology and histologic measures: Expert review. Gastroenterology.

[17] Ladinser B, Rossipal E, Pittschieler K (1994). Endomysium antibodies in coeliac disease: An improved method. Gut.

[18] Kauma S, Kaukinen K, Huhtala H (2019). The phenotype of celiac disease has low concordance between siblings, despite a similar distribution of HLA haplotypes. Nutrients.

[19] Koskinen O, Collin P, Korponay-Szabo I (2008). Gluten-dependent small bowel mucosal transglutaminase 2-specific IgA deposits in overt and mild enteropathy coeliac disease. J. Pediatr. Gastroenterol. Nutr..

[20] Metscher BD (2009). MicroCT for developmental biology: A versatile tool for high-contrast 3D imaging at histological resolutions. Dev. Dyn..

[21] Buades A, Coll B, Morel JM (2005). A non-local algorithm for image denoising. Proc. IEEE Comput. Soc. Conf. Comput. Vis. Pattern Recognit..

[22] Lehmann G, Legland D (2012). Efficient N-dimensional surface estimation using Crofton formula and run-length encoding. Insight J..

[23] Freeman HJ (2008). Pearls and pitfalls in the diagnosis of adult celiac disease. Can. J. Gastroenterol..

[24] Ravelli A, Villanacci V (2012). Tricks of the trade: How to avoid histological pitfalls in celiac disease. Pathol. Res. Pract..

[25] Corazza GR, Villanacci V, Zambelli C (2007). Comparison of the interobserver reproducibility with different histologic criteria used in celiac disease. Clin. Gastroenterol. Hepatol..

[26] Arguelles-Grande C, Tennyson CA, Lewis SK (2012). Variability in small bowel histopathology reporting between different pathology practice settings: Impact on the diagnosis of coeliac disease. J. Clin. Pathol..

[27] Zanini B, Caselani F, Magni A (2013). Celiac disease with mild enteropathy is not mild disease. Clin. Gastroenterol. Hepatol..

[28] Tennyson CA, Simpson S, Lebwohl B (2013). Interest in medical therapy for celiac disease. Therap. Adv. Gastroenterol..

[29] Silvester JA, Kurada S, Szwajcer A (2017). Tests for serum transglutaminase and endomysial antibodies do not detect most patients with celiac disease and persistent villous atrophy on gluten-free diets: A meta-analysis. Gastroenterology.

[30] Sarna VK, Skodje GI, Reims HM (2018). HLA-DQ:gluten tetramer test in blood gives better detection of coeliac patients than biopsy after 14-day gluten challenge. Gut.

[31] Thomas HJ, Ahmad T, Rajaguru C (2009). Contribution of histological, serological, and genetic factors to the clinical heterogeneity of adult-onset coeliac disease. Scand. J. Gastroenterol..

[32] Catassi C, Fabiani E, Fasano A (2007). A prospective, double-blind, placebo-controlled trial to establish a safe gluten threshold for patients with celiac disease. Am. J. Clin. Nutr..

[33] Aziz I, Peerally MF, Barnes JH (2017). The clinical and phenotypical assessment of seronegative villous atrophy; a prospective UK centre experience evaluating 200 adult cases over a 15-year period (2000–2015). Gut.

[34] Juuti-Uusitalo K, Mäki M, Kainulainen H (2007). Gluten affects epithelial differentiation-associated genes in small intestinal mucosa of coeliac patients. Clin. Exp. Immunol..

[35] Myrsky E, Syrjänen M, Korponay-Szabo IR (2009). Altered small-bowel mucosal vascular network in untreated coeliac disease. Scand. J. Gastroenterol..

[36] Schambach SJ, Bag S, Groden C (2010). Vascular imaging in small rodents using micro-CT. Methods.

[37] Holmes R, Hourihane D, Booth C (1961). The mucosa of the small intestine. Postgrad. Med. J..

[38] Loehry CA, Creamer B (1969). Three-dimensional structure of the human small intestinal mucosa in health and disease. Gut.

[39] Kaukinen K, Collin P, Holm K (1999). Wheat starch-containing gluten-free flour products in the treatment of coeliac disease and dermatitis herpetiformis. A long-term follow-up study. Scand. J. Gastroenterol..

[40] Ashton J, West J, Badea C (2015). In vivo small animal micro-CT using nanoparticle contrast agents. Front. Pharmacol..

[41] Easlon H, Bloom A (2014). Easy leaf area: Automated digital image analysis for rapid and accurate measurement of leaf area. Appl. Plant Sci..

[42] Marsh MN (1992). Gluten, major histocompatibility complex, and the small intestine. A molecular and immunobiologic approach to the spectrum of gluten sensitivity ('celiac sprue'). Gastroenterology.

