# Supplementary material for: X-ray microtomography is a novel method for accurate evaluation of small-bowel mucosal morphology and surface area
Source: Sci Rep. 2020 Aug 4;10:13164. doi: 10.1038/s41598-020-69487-w (PMC7403326; doi:10.1038/s41598-020-69487-w)
Supplement: Supplementary file 1 — Supplementary Infomation. [file 41598_2020_69487_MOESM1_ESM.docx]

**Video demonstrations**

**Video 1.** A digital 3D representation of a small-bowel mucosal biopsy was created by reconstructing the original micro-CT imaging data with XMReconstructor 8.1.6599 software (Xradia, Carl Zeiss AG, CA) and the video was produced with Avizo software (Thermo Fisher Scientific, Waltham, MA). The option to freely orientate the digital biopsy and even individual villi for optimal plane of viewing and precise morphometric measurements can be readily seen. The 3D illustration also reveals the highly variable shape of the villi.

**Video 2**. Selection of the plane of measurement. Correct plane of view for the optimal cutting angle was obtained by observing a villus from a cross section that is rotated 180 degrees around an axis placed at the center of the villi. The point where the villus and crypt appear intact at their longest was chosen as the cutting angle. This enabled us to objectively determine the height of the villi and the depth of its adjacent crypts.
