# Supplementary material for: X-ray microtomography is a novel method for accurate evaluation of small-bowel mucosal morphology and surface area
Source: Sci Rep. 2020 Aug 4;10:13164. doi: 10.1038/s41598-020-69487-w (PMC7403326; doi:10.1038/s41598-020-69487-w)
Supplement: Supplementary file 2 — Supplementary Figures. [file 41598_2020_69487_MOESM2_ESM.docx]

**X-ray Microtomography is a Novel Method for Accurate Evaluation of Small-Bowel Mucosal Morphology and Surface Area**

Johannes Virta, Markus Hannula, Ilmari Tamminen, Katri Lindfors, Katri Kaukinen, Alina Popp, Juha Taavela, Päivi Saavalainen, Pauliina Hiltunen, Jari Hyttinen, Kalle Kurppa


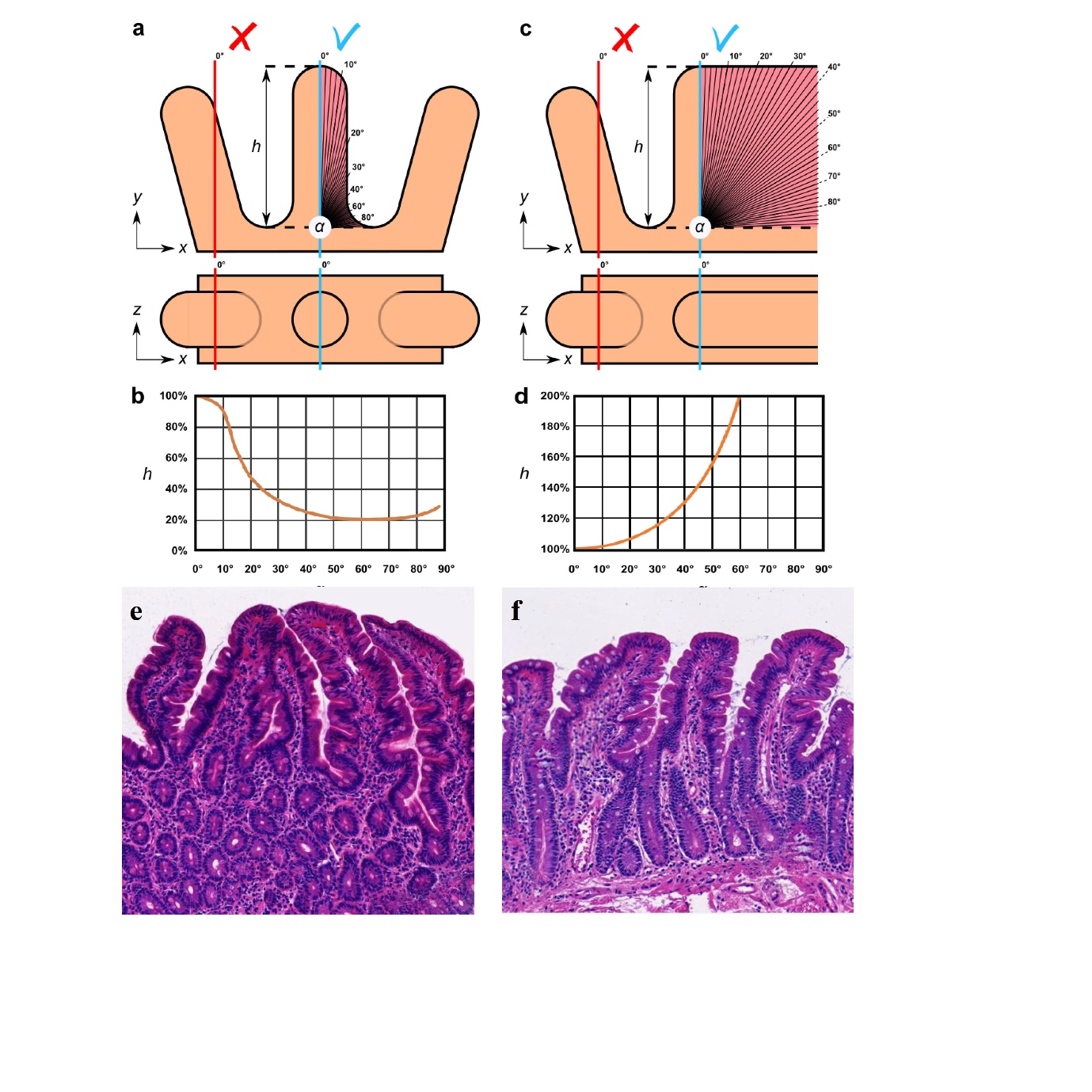


***Supplementary Figure 1***. Theoretical modeling (Panels **a-d**) and real-life example (Panels **e-f**) of the effect of plane of viewing on the diagnostic evaluation of small-bowel mucosal biopsies. Simple geometrical illustrations of finger‑like (**a**) and leaf-like (**c**) villi were used to demonstrate the effect of varying cross‑sectional angles on the apparent height of the villi (**b** and **d** respectively). Blue lines demonstrated in zero angle perfectly aligned with the measured villi. The values in **b** were sampled from the illustrated geometry of **a**, and the values in **d** were obtained with the formula 1/cos(α) * 100%. It is noteworthy that, even with a proper angle for a particular villus, the angle is likely to be suboptimal (red lines) for other villi oriented differently in the same sample, a problem difficult to avoid in conventional histology. The illustrations were drawn in Inkscape 0.91 Vector Graphics Editor^1^ and the graphs in Microsoft Excel 2016 (Microsoft Corp., Redmond, WA). Panels **e** and **f** demonstrate the adverse consequences of suboptimal orientation in the assessment of H&E stained sections in a real patient scenario. Biopsies obtained from an individual with suspected celiac disease show mucosal inflammation, but the villi are seemingly normal and no diagnosis could be established (Panel **e**). Nevertheless, the presence of circular cross-sections of crypts that do not reach the epithelial surface implies incorrect cutting angle and inappropriateness of this section for accurate morphometry. A correctly orientated section from the same patient reveals significantly reduced villous height-crypt depth ratio (Panel **f**).


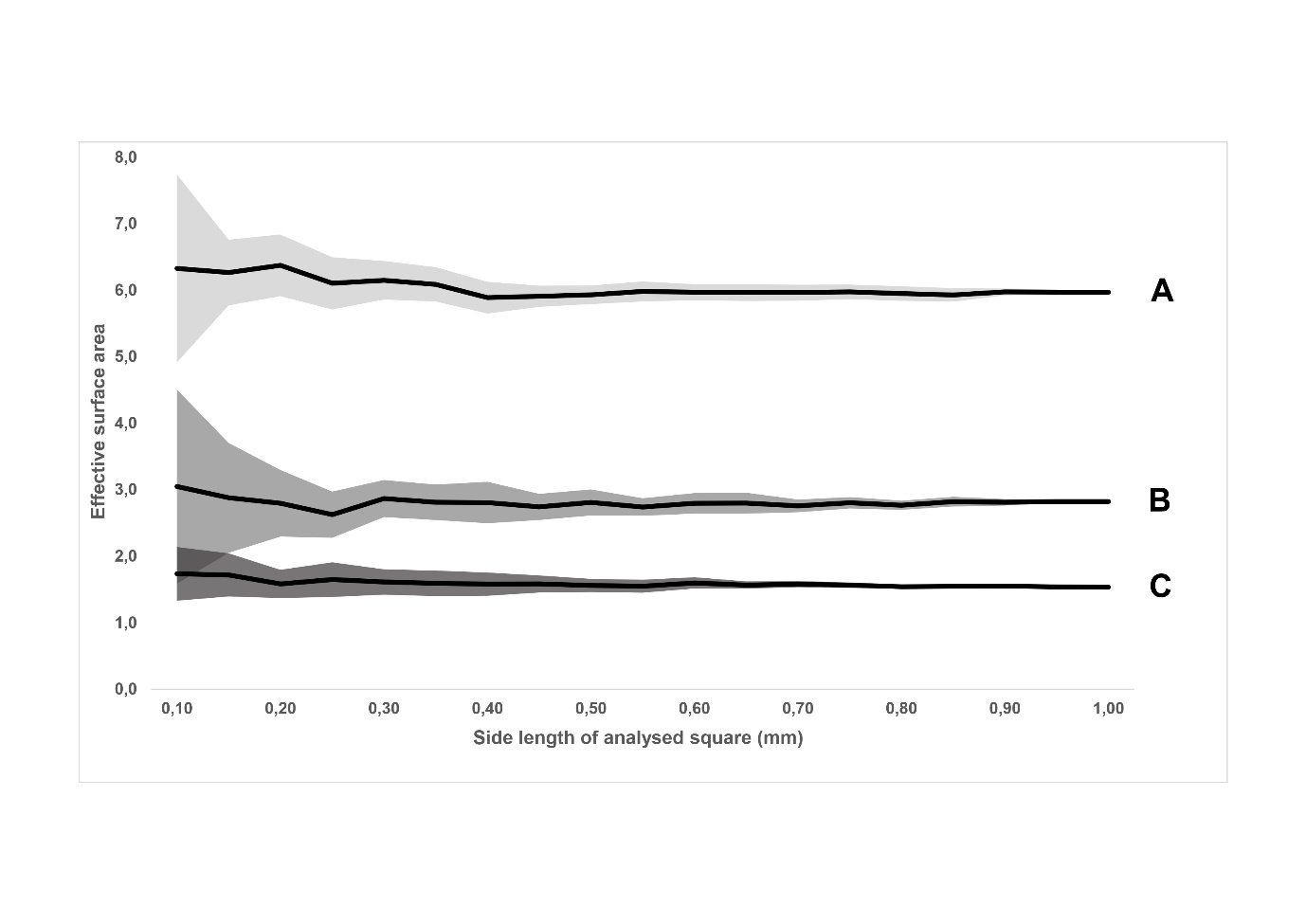


***Supplementary Figure 2.*** Influence of the measurement side length to the variation between repeated surface area measurements applied to the same intestinal biopsy. Squares with increasing side lengths from 0.1 to 1.0 mm at every 0.1 mm were tested by measuring 20 individual measurements from the same biopsy and counting the means and standard deviations. The biopsies were obtained from three subjects having healthy mucosa (A), mild mucosal damage (B) and severe mucosal lesion (C) based on morphometric analysis. The black middle lines denote mean effective surface areas and grey areas standard deviations.

References:

1. Inkscape free online drawing programs for designers. https://inkscape.org/ Accessed 4^th^ June 2019
